# Two-Step Acoustic Cell Separation Based on Cell Size and Acoustic Impedance—toward Isolation of Viable Circulating Tumor Cells

**DOI:** 10.1021/acs.analchem.4c04911

**Published:** 2025-01-17

**Authors:** Cecilia Magnusson, Mahdi Rezayati Charan, Per Augustsson

**Affiliations:** a Department of Translational Medicine, Lund University, Lund SE-22100, Sweden; b Department of Biomedical Engineering, Lund University, Lund SE-223 63, Sweden

## Abstract

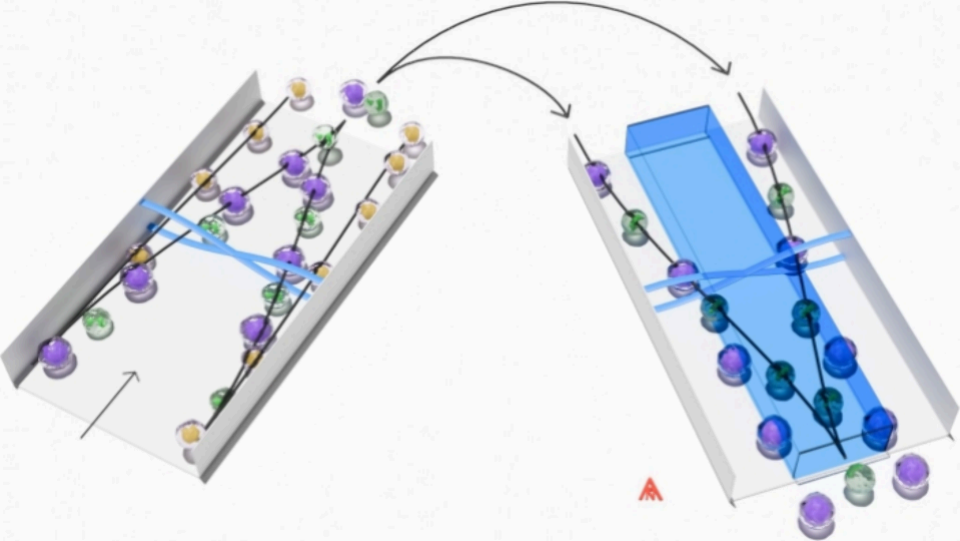

Isolation and characterization of circulating tumor cells (CTCs) present a noninvasive alternative to monitor disease progression in individual patients. However, the heterogeneous lineage specificity of CTCs makes it difficult to isolate and identify possible CTCs by a liquid biopsy. Better label-free methods for the isolation of viable CTCs are needed. Our solution is a combined approach that is inherently epitope independent. Cells are separated by size-sensitive acoustophoresis using an ultrasonic standing wave field, followed by size-insensitive, acoustic barrier-medium focusing, which enables the enrichment of viable cancer cells in blood. With standard acoustophoresis in homogeneous medium, lymphocytes and monocytes were efficiently removed, while removal of granulocytes from the target MCF7 breast cancer cells was not possible due to overlapping acoustic migration velocities for viable cells. Remaining granulocytes were removed by a second separation step with an acoustic impedance barrier-medium selectively blocking the transport of MCF7 cells to generate a clean cancer cell fraction. For two series of 500 mL samples containing 5 × 10^5^ white blood cells, spiked with 2 × 10^4^ or 1 × 10^3^ MCF7 cells, the recovery of MCF7 cells was 77.3% with a 99.9% depletion of white blood cells in the final cancer cell fraction. The most abundant contaminating cell type was granulocytes (85.9% of remaining cells). Nearly all lymphocytes (99.996%) and monocytes (99.995%) were depleted. A two-step acoustic cell separation based on cell size and acoustic impedance is well suited to generate a purified cancer cell fraction as a preparatory step for downstream single-cell analysis.

## Introduction

Circulating tumor cells (CTCs) originate in primary tumors or metastases and are spread to new sites by the peripheral vascular system. It is desirable to capture CTCs with a liquid biopsy;^1^ however, CTCs are extremely rare,^2^ which makes it difficult to isolate them with high purity. Several techniques target surface antigens such as the epithelial cell adhesion molecule (EpCAM), which is exclusively expressed in epithelial cells and epithelium-derived cancer cells. Among these, the CellSearch CTC enumeration system is the most successful and the only US Food and Drug Administration-cleared CTC technology to be used as a prognostic biomarker to predict overall survival in epithelial cancers.^3−5^ The major drawback of this approach is the inability to detect CTCs with a reduced or absent EpCAM expression,^6−8^ e.g., due to an epithelial-to-mesenchymal transition. Such a transition is considered a prerequisite for tumor cell infiltration and metastasis formation at secondary sites.^9,10^ Studies have found that patients with a poor response to chemotherapy had significantly more CTCs of mesenchymal-like phenotype compared to patients who responded well to the treatment.^11−13^ Antibody-based approaches can be highly selective, yet they fail to find many potential subclasses of CTCs, and alternative approaches based on biophysical properties have therefore been developed.

Microfluidic methods to position, separate, and analyze cells hold promise to increase the accuracy and shorten the time from sample to answer in cell-based assays in healthcare and basic biology.^14,15^ Furthermore, microfluidic separation may allow phenotyping of single cells based on their cell-intrinsic biomechanical properties related to differences or changes in their molecular or architectural structure.^16,17^ Properties previously proposed for label-free separation or analysis of CTCs include size,^18^ density,^19^ deformability,^20^ electrical,^21^ and acoustic properties.^22−25^

Acoustofluidic cell separation relies on differences in cell size, density, and compressibility and has been implemented in several different ways. In acoustophoresis, cells flow through a long channel and are exposed to a transverse acoustic standing wave that exerts forces on the cells to deflect them toward the channel center. Large and compact cells are more affected than small and light cells. At the end of the channel, the cells are collected in different outlets at a flow-split. By adding a prefocusing step, the separation accuracy can be considerably improved.^22^ The approach has been shown to be gentle on cells and can be scaled to throughput relevant for CTC enrichment.^23,25^ However, cells are primarily separated by their size, which varies within each cell type, and the overlaps can be large, leading to contaminating white blood cells (WBCs) in the cancer cell fraction. Many separation techniques for CTCs, e.g., CellSearch, use fixed cells. However, to do functional studies or drug testing on the isolated cancer cells, they need to be alive. Unfortunately, the difference in acoustic migration between viable WBCs and cancer cells is even smaller than for paraformaldehyde (PFA) fixed cells,^26^ leading to poor separation outcomes. To improve the specificity of acoustophoresis, an addition of a negative selection step has previously been attempted using elastomeric particles with affinity toward WBCs. This approach generated cancer cell samples with high purity, but the cancer cell recovery was only around 30%.^26^ Combined methods have also been demonstrated, e.g., by separating cells by size followed by depletion of remaining WBCs by magnetic nanoparticles.^27^

In this study, we separate MCF7 breast cancer cells from WBCs using a label-free approach that combines standard acoustophoresis with a second step, wherein cells are separated by isoacoustic focusing. Here, a sound field pushes cells through a gradient medium of increasing acoustic impedance, leading to a size-insensitive spatial separation of cells based on their effective acoustic impedance,^28^ resulting in overall higher cancer cell purity and recovery.

## Methods

### Blood Sample Collection, Cell Culture, and Antibody Staining

Ethylenediaminetetraacetic acid (EDTA)-anticoagulated whole blood (6 mL) was collected in Vacutainer tubes (BD Bioscience, Temse, Belgium) from anonymized healthy volunteers providing signed informed consent at the Biomedical Center, Lund University (Lund, Sweden), according to a protocol approved by the Swedish Ethical Review Authority (ref no. 2020-05818). The breast cancer cell line MCF7 was acquired from ATCC and cultured according to recommendations in a humidified incubator at 37 °C under the influence of 5% CO_2_. Cancer cells were harvested by adding trypsin and used for experiments at around 80% confluency. PFA-fixed WBC samples were prepared by red blood cell (RBC) lysing with 1× BD lysing solution (BD Biosciences) followed by a 20 min incubation with a 4% PFA solution at room temperature with subsequent centrifugation at 400*g* for 5 min. Viable WBCs were prepared by RBC lysing with BD Pharm Lyse (BD Biosciences) followed by centrifugation at 200*g* for 5 min. Both PFA fixed and unfixed cells were stained for 20 min at room temperature with either anti-EpCAM-PE antibody (BD Biosciences) for cancer cell identification, anti-CD45-APC (BD Biosciences) for WBC identification, or anti-CD66b-AF647 (BD Biosciences) for granulocyte identification by flow cytometry (BD FACSCanto II).

### Acoustic Separation Chips and Flow System

**Figure 1:**
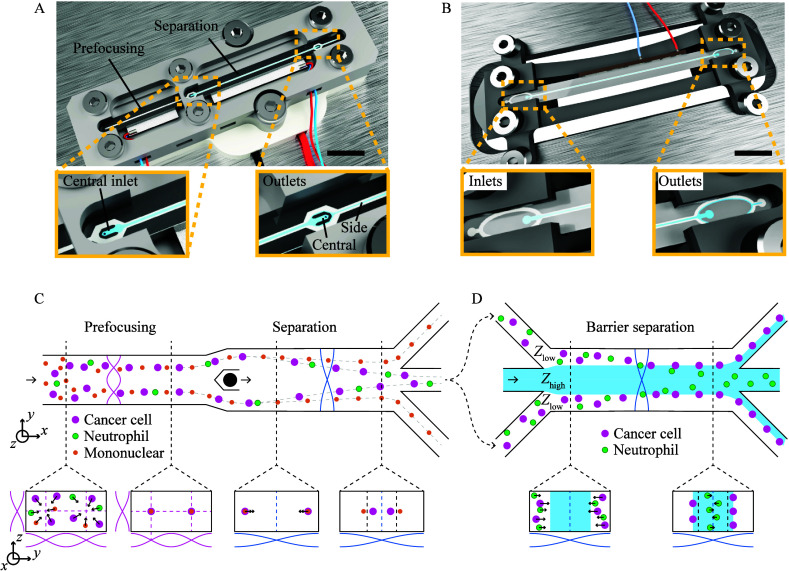
Renderings of the acoustofluidic platforms and cell separation principles. (A) Acoustophoresis chip with prefocusing and a subsequent separation channel. Scale bar: ∼1 cm. (B) Isoacoustic barrier separation chip. Scale bar: ∼1 cm. (C) Schematic of the prefocusing and the subsequent acoustophoresis principle. (D) Schematic of isoacoustic barrier separation based on acoustic impedance (*Z*).

Two different acoustofluidic in-line separation chips were used in this study. The chip used for the primarily size-dependent acoustophoresis cell separation is a silicon-and-glass chip^29^ with two serially linked channels for prefocusing and separation, respectively, which has been previously described^23^ (Figure 1A). The prefocusing channel (length × width × depth: 20 mm × 300 μm × 150 μm) was actuated by using a piezoceramic transducer resonant at ∼4.90 MHz.

The separation channel (30 mm × 380 μm × 150 μm) was actuated by a second piezoceramic transducer resonant at 1.977 MHz. The piezoceramic transducers are both located underneath the chip, and the temperature is controlled by a Peltier element maintaining a constant temperature of 27 °C. The chip used for the acoustic impedance barrier separation is a glass-silicon-glass chip with one separation channel that has been previously described^30^ (Figure 1B). The barrier separation channel has the following dimensions (length × width × depth): 50 mm × 375 μm × 150 μm. The piezoceramic transducer resonant at 1.921 MHz is located on the side of the chip.^31,32^ There is no active temperature control since the side actuation generates less heat than the bottom actuated chips. The flow was driven by a custom pneumatic control system with flow sensor feedback loops that regulate the pressure inside the fluid containers, as previously described.^22^ Photographs of the separation setup is shown in Figure S1.

### Acoustofluidic Separation Principles

Cells were processed through two different chips that can separate cells based primarily on size, as previously shown,^22,23,25^ and on acoustic impedance, as more recently shown.^33^ For step 1 cell separation (Figure 1C), the relative flow rates were set to *Q*_side_^in^ = 20%, *Q*_center_^in^ = 80%, *Q*_center_^out^ = 20%, and *Q*_side_^out^ = 80%, with a total flow rate set to *Q* = 375 μL min^–1^. Cells were infused through the side inlets and first prefocused and levitated to two points in the channel cross section by an acoustic field at 4.900 MHz. Thereafter, the cells were separated by their different acoustically induced migration velocities in a single-node acoustic field. BufferA (1× PBS, 1% FBS, 2 mM EDTA) was used as a suspending medium in the system, unless otherwise specified. Factors that determine the separation are the density, compressibility, and size of the cells. A squared dependency on the size makes cell size the dominating parameter in the separation. Still, cells with a high enough density and low compressibility, such as neutrophils, migrate faster due to a higher acoustic contrast than other cells of similar size. Thus, a comparably small but compact cell can have a similar acoustic migration velocity as a larger but less compact cell such that they end up in the same outlet. In the step 2 isoacoustic barrier cell separation (Figure 1D), the cells encounter a barrier fluid into which only the more compact cells, e.g., granulocytes, can penetrate. In this separation mode, the central flow consists of a medium with an altered acoustic impedance, which prevents cells of low acoustic impedance, e.g., cancer cells, monocytes, and lymphocytes, to penetrate.^28^ The barrier media consisted of BufferA supplemented with various concentrations of OptiPrep (STEMCELL Technologies, Saint-Égrève, France, iodixanol concentration 60%) depending on the desired final iodixanol concentration of the medium. The solution was kept isosmotic by adding a volume of 10× PBS corresponding to 10% of the OptiPrep volume. We used BufferA supplemented with 16% iodixanol as the high-acoustic impedance barrier medium, and for the low-acoustic impedance cell inlet medium, we used BufferA supplemented with 6% iodixanol unless otherwise specified. The flow rate configuration was set to *Q*_side_^in^ = 30%, *Q*_center_^in^ = 70%, *Q*_center_^out^ = 30%, and *Q*_side_^out^ = 70%, with a total flow rate *Q* = 180 μL min^–1^. The voltage amplitude applied to the piezo was established using 3 μm-diameter polystyrene tracer beads by determining the amplitude above in which the band of focused particles started to become distorted by spurious modes of the acoustic field.^34^ At *Q* = 180 μL min^–1^, this occurred above 11.8 V.

The process of transferring the recovered cells from the size-based separation step to the isoacoustic barrier separation required a resuspension of cells in media supplemented with iodixanol by centrifugation and supernatant exchange. It should be noted that this is not a fundamental requirement for the separation mechanism to work but it was done to solve a technical challenge related to the limited capability of the flow sensors in the pneumatic flow control system to cope with the stratified fluid that exits through the side outlet of the barrier separator. To reduce the mismatch and minimize the associated oscillations in the control system, the side inlet medium was elevated to 6% iodixanol.

### Cell Viability after Two-Step Density Gradient Acoustophoresis

An evaluation of long- and short-term cell viability after the two-step acoustofluidic separation was performed by determining cancer cell death. We processed 0.5 mL of cell samples containing 5.0 × 10^5^ MCF7 cells in the size separation chip, followed by a subsequent barrier separation using 16% iodixanol. Samples were run through the system with and without ultrasound to evaluate the effect of ultrasound on cell death. Simultaneously, control cells suspended in either PBS or in 16% iodixanol were incubated on ice throughout the length of the experiment to estimate the effects of either iodixanol exposure or the shear forces in the flow system. For each sample/treatment, 200,000 cells per well were seeded in duplicate six-well plates for subsequent culture. At least 30,000 cells for each sample were analyzed in triplicates by flow cytometry to calculate the concentration of dead cells (7AAD^+^ cells) directly after the experiment and after cell passages 1, 2, and 3. After 3 days, the cells and cell medium (containing dead cells) from the first plate were harvested and the percentage of dead cells was analyzed. Cells (only attached) from the duplicate plates were reseeded in duplicate six-well plates for further passages. The experiment was run in triplicate and repeated two times.

## Results and Discussion

### Separation of PFA-Fixed vs Nonfixed Cancer Cells and WBC

**Figure 2:**
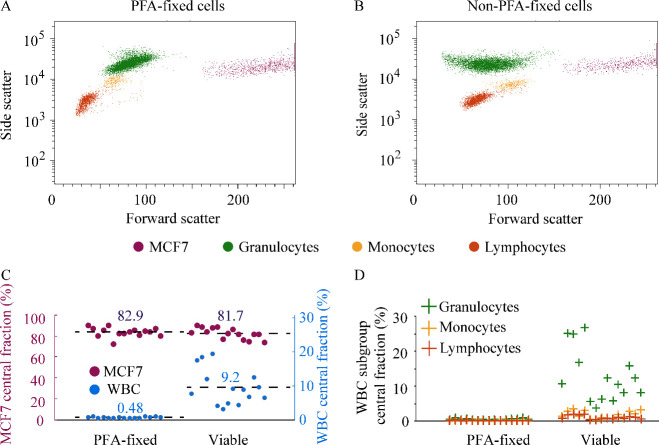
Separation of paraformaldehyde fixed cells versus nonfixed cell separation. Flow cytometer forward/side scatter dot plot of (A) PFA fixed WBC and MCF7 cells and (B) non-PFA fixed cells. (C) Comparison of separation performance by acoustophoresis of PFA-fixed WBCs and MCF7 cells versus nonfixed cells. (D) Separation performance by acoustophoresis of different WBC subgroups for PFA-fixed and nonfixed cells.

We have previously shown that it is more challenging to separate viable prostate cancer cells from WBCs compared to the separation for PFA-fixed cells.^26^ Here, we observe the same trend when MCF7 breast cancer cells are separated from WBCs. For the PFA-treated WBC population, cell size tends to decrease and becomes more uniform, whereas PFA fixation seems to affect the cell size of MCF7 cells to a lesser degree, as shown in Figure 2A,B. Fixation leads to an increased difference in the acoustic migration velocity between the cell types and therefore better acoustic separation. For unfixed cells, allowing at least 80% of the MCF7 cells to focus into the central outlet mediates an almost 20-fold increase of contaminating WBCs compared to fixed cells (Figure 2C). Looking closer at the unfixed WBC populations, it is evident that it is mainly the granulocytes that have similar acoustic properties as the cancer cells and that contaminate the cancer cell fraction collected at the central outlet, Figure 2D.

### Barrier-Medium Acoustic Cell Separation

**Figure 3:**
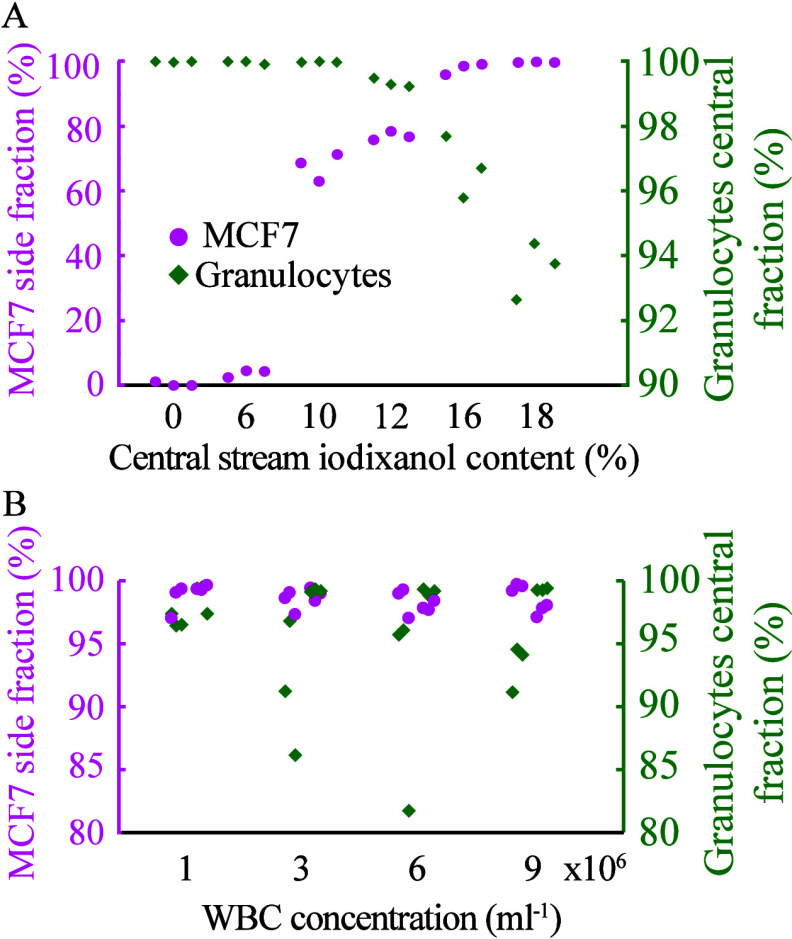
Acoustic medium-barrier separation of white blood cells and cancer cells. (A) Cancer cell and granulocyte separation performance in media of different iodixanol concentrations. (B) Cancer cell and granulocyte separation performance at different cell concentrations.

The overlapping cell sizes and acoustic properties of viable granulocytes and breast cancer cells require a new separation approach. By changing the suspending medium’s acoustic properties, e.g., by altering the medium’s acoustic impedance, the acoustic contrast of a cell can be tuned, and the strong size dependency for cell separation with acoustophoresis can be circumvented.^28,35−37^ When flowing through the channel, all cells initially have positive contrast in their initial medium and move toward the acoustic focusing node. Upon reaching the interface region between the side and central media, only cells that have sufficiently high acoustic impedance can penetrate the central barrier medium, whereas cells with zero or negative contrast in the central medium will stop at the barrier, as shown in Figure 1D. We have previously shown that neutrophils have higher acoustic impedance than MCF7 cells.^28^ To find a suitable barrier medium for which granulocytes would focus to the center, leaving breast cancer cells at the barrier to be collected in the side fraction, we evaluated media of different concentrations of iodixanol to alter the acoustic impedance. Iodixanol is a good alternative since it has a relatively high ratio of acoustic impedance to viscosity, is biocompatible, and can be used for viable cells.^38^ A central medium with 16% iodixanol gave the best separation result for MCF7 cells and granulocytes (Figure 3A). At this iodixanol concentration, the removal of granulocytes started to drop, but still the medium barrier blocked 97.7% of the cancer cells. For lower iodixanol concentrations, almost 100% of the granulocytes will be focused; however, the medium barrier will not be effective enough to block most cancer cells from moving into the center.

### Barrier-Medium Separation Is Independent of Cell Concentration

Size-dependent acoustic cell sorting in a PBS-based medium can maintain maximum separation capacity for WBC concentrations up to 3.4 × 10^6^ cell mL^–1^ due to increasing cell–cell interactions.^23^ For separation with an impedance barrier, the capacity is higher since cells migrate until their equilibrium positions are reached. We investigated cell concentrations up to 9 × 10^6^ WBCs mL^–1^ without observing a decrease in separation performance (Figure 3B). This enables processing of more concentrated samples and thereby smaller sample volumes, leading to faster processing time, important for future patient sample processing.

### Barrier-Medium Separation of MCF7 and White Blood Cells

**Figure 4:**
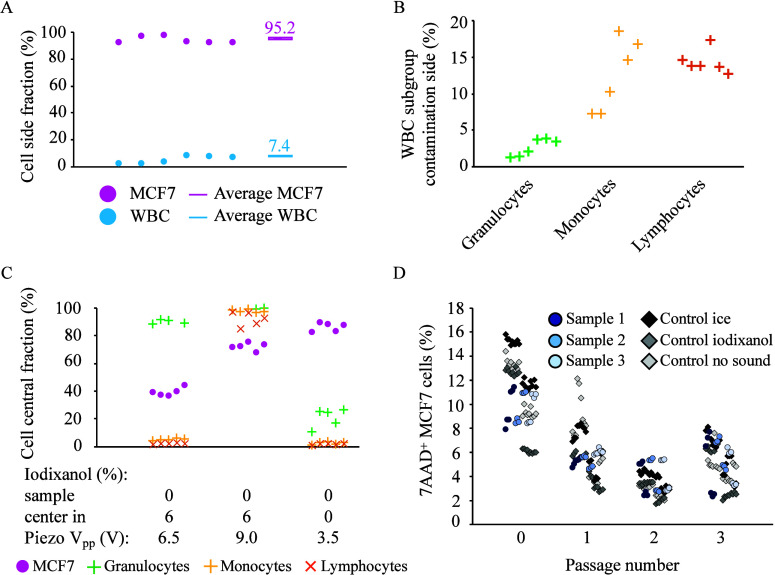
Optimizing settings for the acoustic medium-barrier separation of white blood cells and cancer cells. (A) Acoustic medium-barrier separation of MCF7 cells and WBC with 16% iodixanol as central medium. (B) WBC subgroups from panel (A). (C) Effects of 6% iodixanol as separation buffer on separation of MCF7 cells and WBCs in the size-based separation setup. (D) Effect on cell viability when running a two-step separation.

Separation of MCF7 cells and WBCs using a high-density medium with 16% iodixanol as a barrier generated a cell separation where an average of 95.2% (±2.5%) of the cancer cells were collected through the side outlet together with 7.4% (±2.7%) of the WBCs (Figure 4A). Visualization of the barrier-medium separation revealed that the barrier is steep, but not distinct, due to molecular diffusion and that granulocytes but not MCF7 cells could penetrate the central flow (Figure S2). A closer look at the WBC subtypes revealed that it is mainly monocytes and lymphocytes contaminating the cancer cell fraction (Figure 4B), whereas in the size-based separation, granulocytes were the major contaminant. This indicates that a combined separation approach can lead to improved separation. The barrier separation requires a sample medium with 6% iodixanol to avoid oscillations in the pneumatic flow control circuit. The size-based separation has previously been run in an iodixanol-free medium. To avoid an intermediate centrifugation step to condition the medium for barrier separation, we investigated the possibility of performing size-based separation directly in media containing 6% iodixanol. However, this led to smaller differences in acoustic contrast between the cells resulting in dramatically decreased MCF7 recovery and WBC depletion (Figure 4C). Full integration will thus require an improved flow control system.

### Barrier-Medium Acoustic Separation Does Not Affect Cell Viability

We have previously shown that acoustophoresis does not negatively affect the cancer cells and is a very gentle separation method.^26,39^ For our proposed two-step separation approach, we investigated how adding iodixanol and multiple separation steps affects the cells both for short and long terms by looking at cell viability directly after a two-step acoustophoresis cell separation and after up to three cell passages. Control cells incubated on ice during the length of the experiment showed that the effect of being out of cell culture for a couple of hours had a significant impact on cell viability. However, neither incubation in iodixanol nor the acoustic separation steps showed any significant additional effect on cell viability, as shown in Figure 4D. This demonstrates the importance of minimizing the time that the cells are out of culture and, for patient samples, minimizing the time from blood draw to cell culture. It has previously been reported that exposure of cells to media containing agents that increase the density, such as iodixanol (OptiPrep), polysaccharides (Ficoll), or polymer-coated colloidal silica particles (Percoll), is associated with altered properties and function.^40−43^ Therefore, prolonged exposure at high concentrations should be avoided, even though cell viability might not be significantly affected.

### Two-Step Acoustic Cell Separation Based on Cell Size and Acoustic Impedance

**Figure 5:**
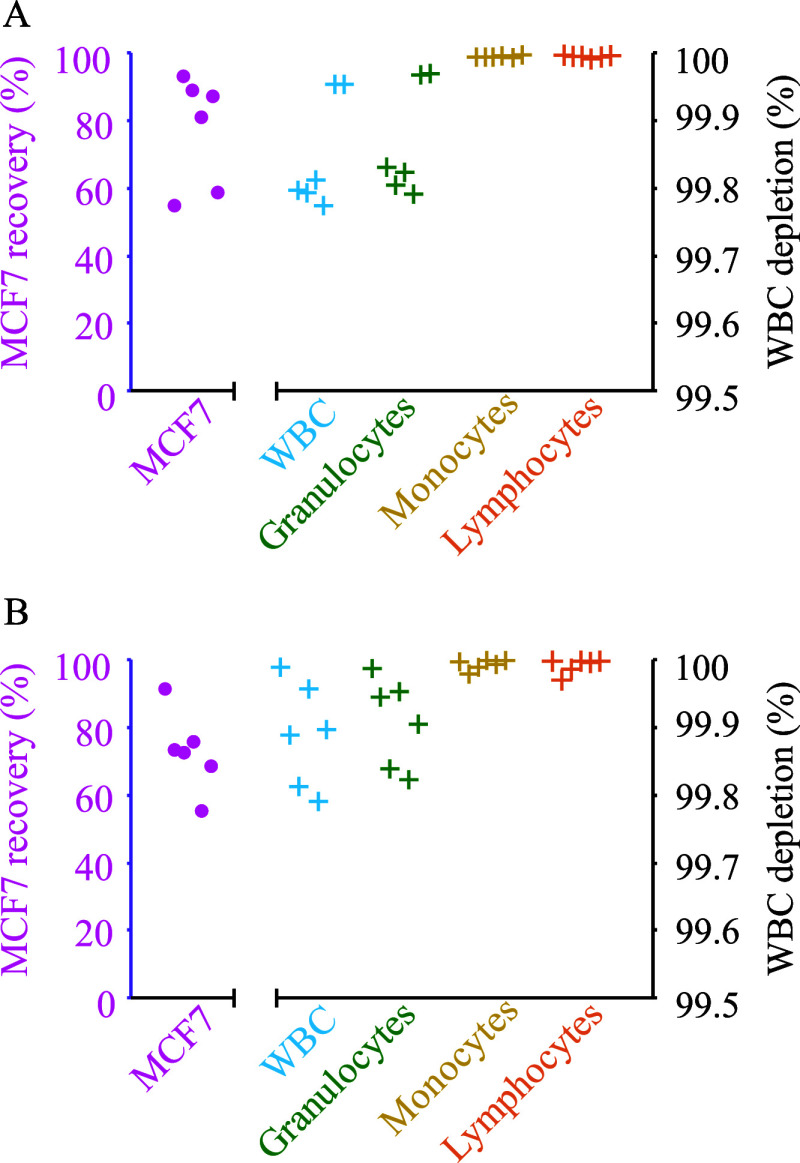
Two-step separation of white blood cells and cancer cells. Six samples (0.5 mL) with 500,000 WBCs spiked with either (A) 20,000 MCF7 cells or (B) 1000 MCF7 cells. Samples were run through first size-based acoustophoresis followed by medium-barrier acoustic separation.

We performed a sequence of size-based separation followed by barrier-medium separation for samples (0.5 mL) containing 5 × 10^5^ WBCs spiked with 2 × 10^4^ MCF7 cells (Figure 5A) or 1 × 10^3^ MCF7 cells (Figure 5B), respectively. The recovery of MCF7 cells were 77.3% (10,936 to 18,614 cells) and 72.9% (553 to 914 cells), respectively, with a 99.9% depletion of WBCs in the collected side outlet fraction after step 2. Granulocytes were the most abundant contaminating cell type, representing 85.9% of the remaining WBCs. However, only 0.013 and 0.009% of the granulocytes from the respective initial samples remained after the separation steps. Additionally, the two-step separation depleted almost all lymphocytes (99,996%) and monocytes (99.995%). The loss of cancer cells can mainly be attributed to incomplete focusing of cells to the central outlet in the first size-based separation step. However, cell loss also occurs in test tubes during the washing between the separation steps. For WBC subtype distribution in the different input and output samples, see Table S1. This approach is an improvement compared to our previous two-step approach, where we used elastomeric negative acoustic contrast particles as a second step to purge the cancer cell fraction from contaminating WBCs.^26^ We observed 2.6- to 2.75-fold increases in cancer cell recovery compared to the previous approach. In addition, the cancer cell fraction is less contaminated with WBCs (0.1% vs the previous 0.4%). Apart from the better performance in terms of cancer cell enrichment and WBC depletion, the current approach is label-free and thus less costly, less laborious, and faster. In addition, the density medium purging step can process higher cell concentrations, allowing for less restrictive conditions in the first size-based separation step. This allows smaller cancer cells to be focused together with higher contamination levels of granulocytes, which can subsequently be removed by the impedance separation step. Our previous two-step separation method^26^ had a contamination cutoff of around 1.5 million WBCs per mL sample after the first step due to clogging of the granulocyte-particle clusters formed in the negative selection in step 2.

## Conclusions

There is no evidence of early detection of disease by monitoring CTCs; however, there is great potential in the areas of disease monitoring and personalized medicine. The possibility of enriching viable CTCs from blood from cancer patients opens the possibility of cell culturing and drug susceptibility testing after mutations as well as fundamental studies of cancer biology. Our work shows that a two-step acoustic cell separation based on cell size and acoustic impedance is well suited to produce a purified cancer cell fraction as a preparatory step for downstream single-cell analysis. The flexibility of the method makes it easy to operate and adapt to different requirements. This proof-of-concept study was limited to one cell line, and our continued efforts will be focused on testing the feasibility of the method for a broader panel of cell lines at realistic concentrations and a subsequent transition to validate the method for enrichment of viable CTCs from blood of cancer patients. Furthermore, we think the specificity and the generality of the platform for targeting different cell types has potential for improvement by incorporating more branches at the outlets of the two chips and by implementing a less steep transition of the barrier medium such as is the case in isoacoustic focusing.^28^

## Supporting Information Available

Complementary overview photos of the setup (Figure S1), photos of granulocytes focusing into the central barrier medium (Figure S2), and tabulated data of the counted cells in the input and output samples (Table S1) (PDF)
