# Supplementary material for: Two-Step Acoustic Cell Separation Based on Cell Size and Acoustic Impedance—toward Isolation of Viable Circulating Tumor Cells
Source: Anal Chem. 2025 Jan 17;97(4):2120–6. doi: 10.1021/acs.analchem.4c04911 (PMC11800186; doi:10.1021/acs.analchem.4c04911)
Supplement: Supplementary file 1 — ac4c04911_si_001.pdf [file ac4c04911_si_001.pdf]

Supporting information

Two-step acoustic cell separation based on cell size and acoustic impedance – Towards isolation of viable circulating tumor cells

Cecilia Magnusson[a], Mahdi Rezayati Charan[b], Per Augustsson[b]*

[a] Department of Translational Medicine, Lund University, Lund SE-22100, Sweden.

[b] Department of Biomedical Engineering, Lund University, Lund SE-223 63, Sweden.

*Corresponding author: Per Augustsson, Email: per.augustsson@bme.lth.se

Table of contents Page

S1. Acoustic microfluidic separation system s2

S2. Barrier focusing s3

S3. Cells in input and outlet samples s4

S1. Acoustic microfluidic separation system

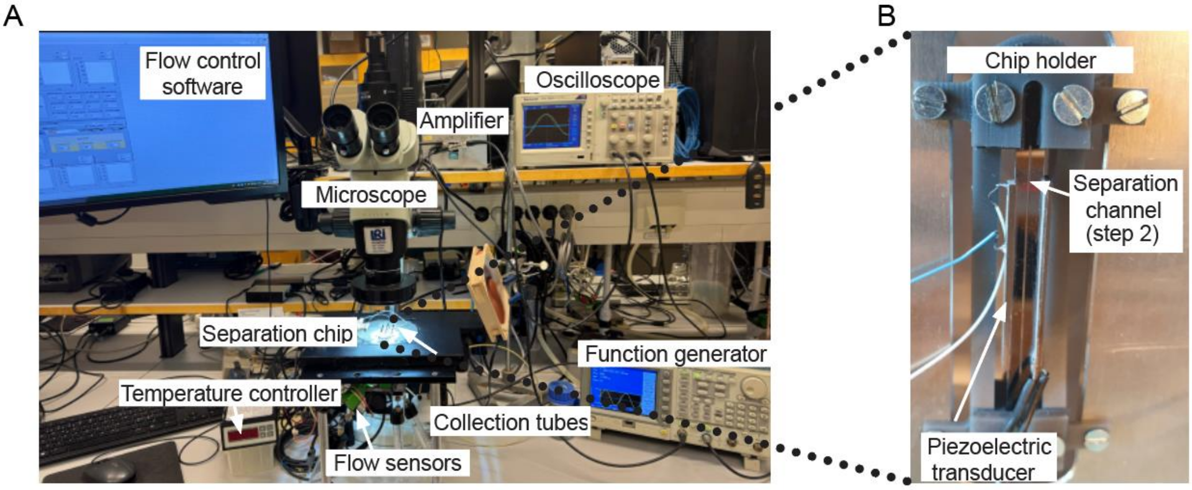

Figure S1. An overview of the acoustic microfluidic separation system. (A) The photo shows the different components of the acoustic separation setup. (B) The step 2 barrier separation chip in its holder.

S2. Barrier focusing

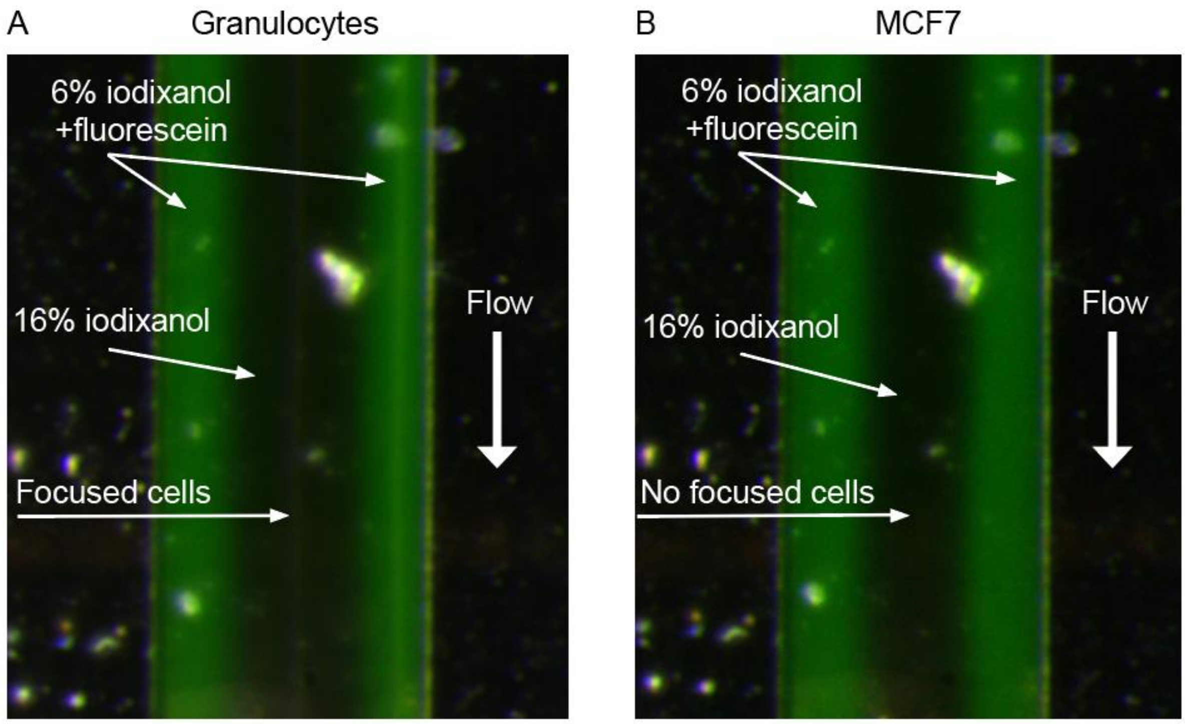

Figure S2. Barrier focusing of granulocytes and MCF7 cells. Separate samples of unstained (A) granulocytes and (B) MCF7 cells processed through the density barrier chip. The side/sample media contains 6% iodixanol supplemented with fluorescein (green). The central medium contains 16% iodixanol and no fluorescein.

S3. Cells in input and outlet samples

Table S1. Number of cells in input samples and side outlet samples in 2-step separation

Sample	MCF7	WBC			Granulocytes			Monocytes			Lymphocytes		
#	Input	Input	Output	Depletion	Input	Output	Depletion	Input	Output	Depletion	Input	Output	Depletion
1	20000	500000	1019	0,002038	344000	836	0,00243	19000	28	0,00147	119000	13	0,00011
2	20000	500000	1031	0,002062	344000	972	0,00283	19000	23	0,00121	119000	20	0,00017
3	20000	500000	941	0,001882	344000	877	0,00255	19000	29	0,00153	119000	18	0,00015
4	20000	500000	1130	0,00226	344000	1040	0,00302	19000	14	0,00074	119000	34	0,00029
5	20000	500000	239	0,000478	298500	156	0,00052	32000	36	0,00113	125000	27	0,00022
6	20000	500000	234	0,000468	337000	155	0,00046	44000	13	0,00030	105500	14	0,00013
1	1000	500000	46	0,000092	381500	53	0,00014	23500	2	0,00009	83000	1	0,00001
2	1000	500000	553	0,001106	381500	264	0,00069	23500	99	0,00421	83000	146	0,00176
3	1000	500000	934	0,001868	381500	801	0,00210	23500	41	0,00174	83000	58	0,00070
4	1000	500000	207	0,000414	381500	224	0,00059	23500	0	0,00000	83000	2	0,00002
5	1000	500000	1043	0,002086	317000	880	0,00278	42000	21	0,00050	123500	8	0,00006
6	1000	500000	512	0,001024	317000	467	0,00147	42000	0	0,00000	123500	3	0,00002
